# Quantifying RNA Degradation with Single-Molecule Nanopore Sensing

**DOI:** 10.1021/acs.analchem.5c03019

**Published:** 2025-10-23

**Authors:** Max K. Earle, Mohammed Alawami, Raluca-Elena Alexii, Simon Brauburger, Ulrich F. Keyser, Casey M. Platnich

**Affiliations:** Cavendish Laboratory, 2152 University of Cambridge, JJ Thomson Avenue, Cambridge CB3 0HE, U.K.

## Abstract

RNA is the key biomolecule in innumerable diagnostic and therapeutic applications, but its chemical instability plagues researchers and clinicians alike. Gel electrophoresis remains the predominant method for the assessment of RNA degradation. The main drawback is the quantity of RNA required—typically 100 ng or more. To study the degradation profiles of mRNA vaccines, viral and bacterial RNA, and other valuable species, new sensitive and quantitative methodologies are required. We present the use of solid-state nanopore sensing to evaluate the degradation of viral RNA under various conditions with single-molecule resolution. While relying on similar principles to gel electrophoresis, nanopore sensing is suitable for use over a wide range of concentration regimes, with even 100 pg of RNA being sufficient for analysis. Our results demonstrate the utility of nanopore assays in assessing RNA integrity for samples not suitable for gel-based analyses due to low abundance or high molecular weight.

## Graphical abstract

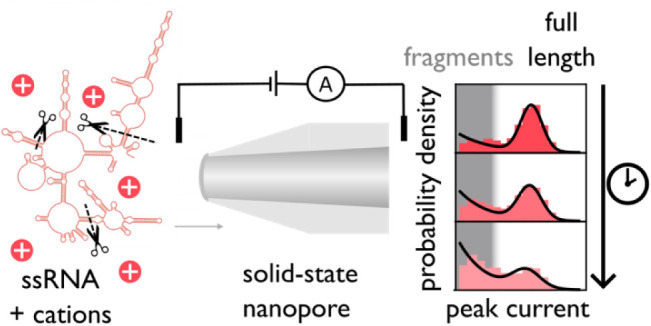

## Introduction

The quantitative detection of RNA molecules is essential toward disease diagnosis, prognosis, and treatment.^1^ Several technologies now exist to detect specific RNAs within biological samples, including next-generation RNA sequencing (RNA-seq),^1–4^ microarray detection,^5–7^ and reverse transcription quantitative polymerase chain reaction (RT-qPCR).^8–10^ These methods all involve the hybridization of the target RNA to some complement strand (either a primer or a probe strand). To enable this hybridization, the RNA’s native secondary structure must be disrupted, typically through a thermal annealing protocol. Furthermore, for PCR, the reaction mixture is usually held at an elevated temperature of 70–75 °C to allow polymerase enzymes to extend the primers. Unfortunately, these necessary heating procedures may also contribute to RNA degradation.^11,12^

The RNA may be attacked through multiple concomitant pathways. Ribonucleases (RNases) are ubiquitous and can degrade RNA at rates as high as 39 nmol/min per mg.^13^ RNAs are also sensitive to oxidation by reactive oxygen species.^11^ Frustratingly, even under controlled reaction conditions wherein RNases and reactive oxygen species are excluded, the phosphodiester linkage can be broken through transesterification, a process often referred to as RNA self-cleavage or autohydrolysis. The reaction proceeds when the 2’OH initiates a nucleophilic attack on a neighboring phosphorus atom, cleaving the P–O bond, with the reaction catalyzed by both acidic and basic conditions. This cleavage behavior is intrinsic to RNA and is the main pathway for the uncatalyzed degradation of RNA in normal cellular conditions.^14^ Self-cleavage is also essential for RNA splicing. The autohydrolysis of RNA is highly dependent on local secondary and tertiary structure due to geometrical constraints in the transition state.^15^ As such, the rate of cleavage varies by up to 10,000 fold with both RNA sequence and reaction conditions including pH, temperature, and salt.^11,16^ It is therefore essential to quantify RNA self-cleavage under different conditions to optimize the state-of-the-art technologies available for RNA detection and RNA-based diagnostics.

RNA degradation has been studied by a variety of methods. In most laboratories, RNA integrity is assessed by benchtop gel electrophoresis (agarose or polyacrylamide, depending on RNA size). Uncompromised RNA gives tight bands, while degraded RNA results in a smear or distinct, higher mobility degradation products. While prized for its simplicity and low cost,^17^ this method requires large quantities of material due to low sensitivity—on the order of 100 ng—and accurate quantification is impossible. Sensitive electrophoresis techniques such as capillary gel electrophoresis achieve improved quantification accuracy with less material, but still require more than 15 picograms of material, have limited accuracy and instrumentation is expensive and bulky.^18^ A fundamental limitation on the study of RNA degradation by electrophoresis is the reliance on fluorescent nucleic-acid staining dyes. The fluorescence intensity of a dyed ssRNA of a certain length could differ by as much as 50%, depending on the RNA sequence in question and its structure.^19^ This goes even for high-performance intercalating dyes such as SYBR Gold and SYBR Green.^20,21^ Very accurate and precise ssRNA degradation studies have been carried out using RT-qPCR with primers designed to produce amplicons of various sizes, but this approach requires the design and synthesis of primers specific to the RNA of interest.^22,23^

In this work, we employ nanopore sensing to quantitatively assess the self-cleavage of RNA under various conditions, demonstrating that studies of RNA degradation may be conducted at the single-molecule level. Nanopore experiments only require down to approximately one picogram of MS2 RNA^24^ making this methodology suitable for the analysis of RNA cleavage events in low abundance samples, including clinically relevant ribozymes or RNA vaccines.^25,26^ Furthermore, this technique is label and primer-free, so that it can be used directly with any ssRNA of known or unknown sequence. In contrast to the mass-based quantification of electrophoresis, nanopore sensing also allows the direct counting of RNA fragments. Every fragment detected by the nanopore can be measured and used to build up a picture of RNA degradation at the single-molecule level.

Nanopore sensing provides a wealth of information on RNA structure, as demonstrated in previous reports wherein solid-state nanopores have been used to assess the secondary structure of viral RNA,^27^ to observe tRNA conformational dynamics,^28,29^ and to detect specific microRNAs.^30,31^ RNA homopolymers have also been studied using solid-state nanopores, revealing that purine and pyrimidine bases may be discriminated using these methods.^32,33^ In previous work from our own group, the Watson–Crick–Franklin base pairing between RNA sequences of interest and DNA complements was leveraged to reshape RNA.^12^ Herein, we examine RNA in its native secondary structure, directly examining the size of these structures with solid-state nanopore sensing. The MS2 viral RNA was selected as its length (3.6 kB) is comparable to many mRNAs with diagnostic and therapeutic value. For example, the mRNA used in COVID-19 vaccine formulation is 3.8 kB. The MS2 RNA used is purchased from Roche and sold as an analytical standard; We aliquoted this material immediately upon receipt and stored it at −80 °C to ensure the RNA remains as undegraded as possible. Throughout this work, this will be referred to as “untreated RNA”. It is important to note, however, that these biological systems are inherently heterogeneous and some RNA degradation during handling/storage is inevitable.

## Experimental Procedures

### Nanopore Sensing

Measurements are conducted using quartz glass nanopores,10–15 nm in diameter. The nanopore separates two chambers containing an aqueous ionic solution and a single-stranded viral RNA (ssRNA, 3.6 kB) sample. A voltage is then applied across the nanopore, driving ions through the pore according to their charge (Figure 1A) while the current through the pore is measured. The RNA, being negatively charged, is electrophoretically pulled through the nanopore, causing a temporary blockage that yields a measurable current drop (Figure 1B).

**Figure 1:**
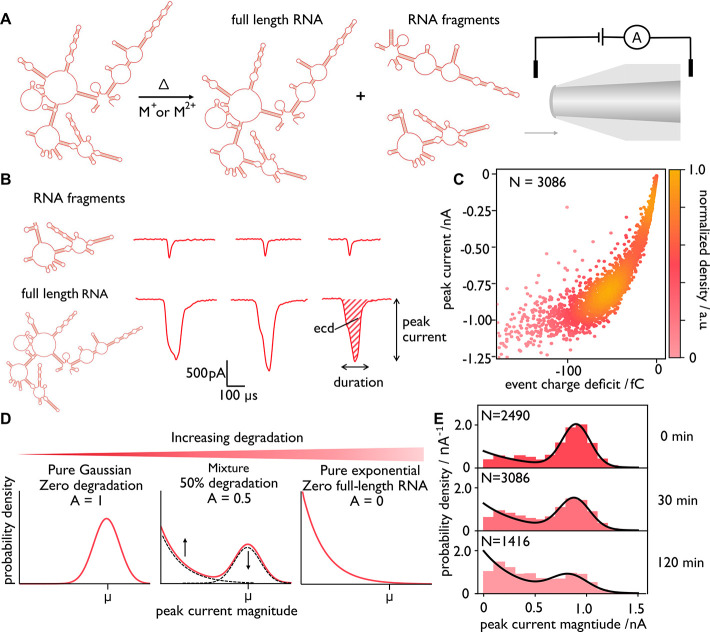
Overview of RNA analysis using solid-state nanopores. (A) When RNA is heated in the presence of salts (with M^+^ or M^2+^ cations, in our case), self-cleavage may occur. The resulting fragments, as well as the full-length RNA, can be detected via nanopore sensing. The RNA shown is only to illustrate the idea and does not depict the actual MS2 sequence or its self-cleavage products. (B) Sample events for RNA fragments and full-length RNA. (C) Scatter plot of peak event current as a function of event charge deficit (ECD) for all events (*N* = 3086) in a single nanopore experiment (here MS2 RNA heated to 70 °C for 30 min in water containing tris buffer only, no salt, pH = 8.0). (D) In an ideal case with a completely undegraded sample, the population of peak currents is Gaussian, centered at the mean, μ. As degradation occurs, the population shifts toward lower peak currents until a purely exponential population exists. Using this analysis, the populations of full-length RNA and RNA fragments may be quantified, enabling the comparison of different experimental conditions. (E) Probability density plots for MS2 RNA heated to 70 °C for 0, 30, and 120 min in water containing tris buffer only, no salt, pH = 8.0. The exponential contribution is scaled based on the Gaussian contribution so that each plot is normalized to an area of 1.

Each nanopore translocation event may be described using two main parameters, namely the peak depth and duration of the current drop. Both parameters are related to the length of the RNA strand that has translocated the nanopore. While the area of the event (also known as the event charge deficit, ECD) is often used as a proxy for molecular weight, we find that the peak current may be more effective for differentiating single-stranded RNAs of differing length (Figure 1C). As shown in Figure 1C, examination of the peak current facilitates the distinction between populations of full-length versus shortened RNAs. We posit that peak current is a more useful measure than ECD in this case because (a) ssRNA exhibits heterogeneous dwell times in the pore due to interactions between the hydrophobic nucleobases and the pore walls and (b) the ssRNA is highly structured and therefore translocations appear as spikes rather than elongated, single-file events. The Actis group have also shown that peak current may be used to readily distinguish between different lengths of single-stranded RNA;^27^ in their case, variable lengths of the Chikungunya virus were generated using T7 RNA polymerase and detected with glass nanopores. Figure S1 shows similar results from our lab demonstrating that ssRNA species of different sizes can be distinguished by peak current magnitude even within the same sample. Using this measurement and analysis strategy, solid-state nanopore sensing can be thought of as the single-molecule equivalent of gel electrophoresis. Importantly, solid-state nanopore techniques can be used to analyze RNA species at the picomolar level,^24^ decreasing the quantity of material required by approximately 3 orders of magnitude relative to gel-based approaches.

In an ideal case, the untreated RNA would be homogeneous in length and therefore yield a single population of deep current blockades. Due to RNA’s conformational flexibility, we would expect the population of peak currents to be normally distributed (Figure 1D). When this full-length RNA is fragmented, the population of peak current becomes more heterogeneous with the emergence of shallower signals, as depicted in Figure 1B. We expect the length distribution of degraded molecules to be approximately exponential, assuming that cleavage is stochastic. As a result of this behavior, the population of peak current magnitudes shifts to greater values while the fragments increase in abundance, as shown in Figure 1D. By quantifying the relative areas of these two contributions, we can determine the amount of degradation that has occurred. A proof-of-concept with intentionally sheared M13 DNA is shown in Figure S2 and demonstrates how populations of peak currents shift in response to forced degradation. We note that some exponential character is observed even at *T* = 0. We conclude that some fragmented nucleic acids will always be present, even in an untreated sample, as a result of synthesis, purification, long-term storage (even at −80 °C) and inevitable sample preparation steps. Measurement of the pure MS2 matrix buffer (10 mM Tris-HCl, 1 mM EDTA, pH 7.0) without RNA returns a very low rate of transient events due to noise, with no distinct subpopulations. Therefore, the background of these measurements is very low and can be ignored (Figure S3).

### Model Fitting

The model to fit the experimentally observed distributions based on peak currents values is depicted in Figure 1D. The model takes four parameters: the Gaussian scaling coefficient, the Gaussian mean, the Gaussian standard deviation and the exponential decay constant. Here, the key parameter is the Gaussian scaling coefficient, which corresponds to the percentage of full-length RNA molecules present in the sample. Fitting was carried out via maximum likelihood estimation (MLE), with the model and fitting procedure further described in the Supporting Information.

Our approach has three major strengths. First, degradation is not a binary process and there is a continuum of resulting fragment sizes, including some fragments only slightly shorter than the untreated RNA. As such, the distribution of peak depths of the degraded RNA may overlap with the Gaussian distribution corresponding to the full-length molecules. This is especially true considering the conformational flexibility of the ssRNA, which leads to a distribution of peak depth values even for molecules of equal length. By fitting to the whole distribution, our approach enables us to estimate the proportion of undegraded RNA even in regions of overlap. Second, fitting to the distribution of the degraded RNA allows us to estimate the proportion of molecules smaller than the nanopore detection threshold. The fitted model does not go to 0 near the origin, even though the histogram drops off markedly as the fragments are too small to be detected. This is an advantageous feature of fitting our model by maximum likelihood estimation rather than least-squares regression to a histogram or kernel density estimate; regions where fewer observations are made than expected do not directly suppress the model fit if the rest of the data fits well.^34^ At peak current values below the nanopore’s threshold, the nonzero value of the probability distribution model amounts to an estimation of the proportion of undetected fragments. Because of the normalization, the estimate of intact RNA is reduced in accordance with the estimate of fragments below the detection limit. This mitigates the skewing effect of leaving highly degraded species uncounted, reducing uncertainty, especially for very degraded samples.

### RNA Handling

In this work, we use all RNase-free reagents and work using RNA-safe protocols to minimize contamination by RNases. As such, we assume that self-cleavage is the main pathway for RNA degradation. While computational studies have interrogated the impact of RNA length and sequence on degradation,^35^ these models often do not take into account cation identity or charge. We investigate the impact of these variables on RNA degradation and demonstrate that full-length RNA may be distinguished directly from its degradation products using solid-state nanopore sensing. By varying the duration of the heating step, as well as the identity and charge of the cations, we tested a matrix of possible procedures and quantified the resulting degradation in each case. The comparison of agarose gel electrophoresis (Figure S5) assays with solid-state nanopore measurements highlights the similarities in results afforded by these two methods while underlining the advantages of nanopore sensing, as the amount of sample needed is decreased by ∼1000 fold. Our results thus demonstrate the utility of nanopore assays in assessing RNA integrity for samples not suitable for gel-based analyses due to low abundance.

## Results and Discussion

### RNA Degradation at 70 °C with Monovalent Salts

We began by examining RNA degradation in 10 mM tris buffer at pH 8.0 (Figure 1C,E). Prior to loading the RNA sample into the nanopore chip, we simulated common PCR protocols by heating the RNA to 70 °C (as in the primer extension step of PCR) using a thermocycler. At the end of the heating interval, the RNA was immediately placed in an ice bath and diluted in 4 M LiCl for nanopore detection. The sample was then directly characterized using solid-state nanopore sensing, to limit any further degradation. The resulting current–time trajectories were analyzed using a custom Python script to determine the peak current value of each event. Doing so for hundreds of individual events revealed populations of deeper events, corresponding to full-length MS2, as well as shallower events, which we ascribe to RNA fragments (Figure 1E), commensurate with agarose gel electrophoresis results (Figure S5). As shown in Figure 1E at *T* = 0 min, a subpopulation of short RNA species is observed, indicating that some degradation products are already present even when untreated MS2 RNA is taken directly from −80 °C storage. Notwithstanding this initial heterogeneity, these experiments reveal that longer incubation times at 70 °C result in increased RNA degradation (Figure 1E), with a shift toward lower peak currents. While this finding is expected, it demonstrates that nanopore experiments can report on RNA degradation.

Following on, we tested the influence of both divalent and monovalent cations on RNA degradation. To facilitate comparison, the pH was held constant at 8.0 using 10 mM tris buffer. For monovalent cations, we selected Li^+^, Na^+^, and K^+^ to probe RNA degradation (Figure 2). Li^+^ is among the preferred cations in the solid-state nanopore community, prized for its ability to effectively slow the translocation of DNA and RNA,^36^ offering higher resolution. On the other hand, Na^+^ and K^+^ are used in the field of DNA/RNA nanotechnology to fold complex structures, including origami.^37,38^ As for the tris buffer only (no salt) experiments, the RNA was heated to 70 °C in a thermocycler for a given time interval in 10 mM tris buffer (pH 8.0) and 100 mM of LiCl, NaCl, or KCl in nuclease-free water. For all conditions, the peak current probability densities shift from an initial, predominantly Gaussian form at higher peak currents to an exponential population at lower peak currents as degradation occurs. Furthermore, there is a tendency for the center of the Gaussian peak to drift toward smaller peak current magnitudes reflecting degradation from ssRNA ends, (Figure S6) although this is relatively minor compared to the reduction in size of the peak.

**Figure 2:**
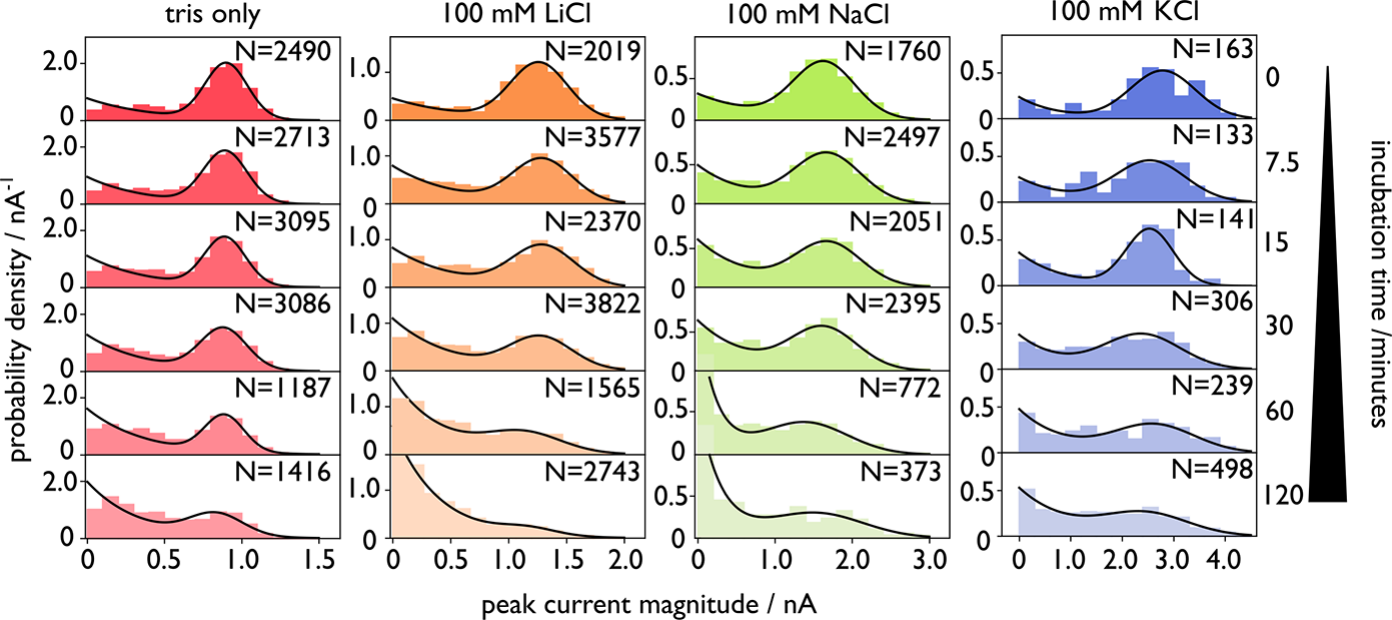
Histogram of single-molecule peak currents from nanopore experiments for MS2 RNA degradation over time. Samples were heated to 70 °C in nuclease-free water with 10 mM tris buffer (pH 8.0) and either no salt (leftmost panel) or 100 mM monovalent salt (LiCl, NaCl, or KCl, left to right). With increased heating time as shown in each panel, the populations of peak currents shift from the dominant Gaussian (corresponding to the full-length construct) to an exponential tail, indicating degradation. The exponential contribution is scaled based on the Gaussian contribution so that each plot is normalized to an area of 1 to conserve probability.

Using our maximum likelihood estimation from the peak current values, we determine the percentage of full-length RNA at each time point in our various salt conditions. We assume that the proportion of full-length molecules decreases approximately exponentially with time, as shown in Figure 3. An exponential function was thus fitted to the data for full-length proportion against time using least-squares regression. The resulting degradation rate constants and derived half-lives can then be used to compare the rate of self-cleavage across different conditions (Figure 3A–D).

**Figure 3:**
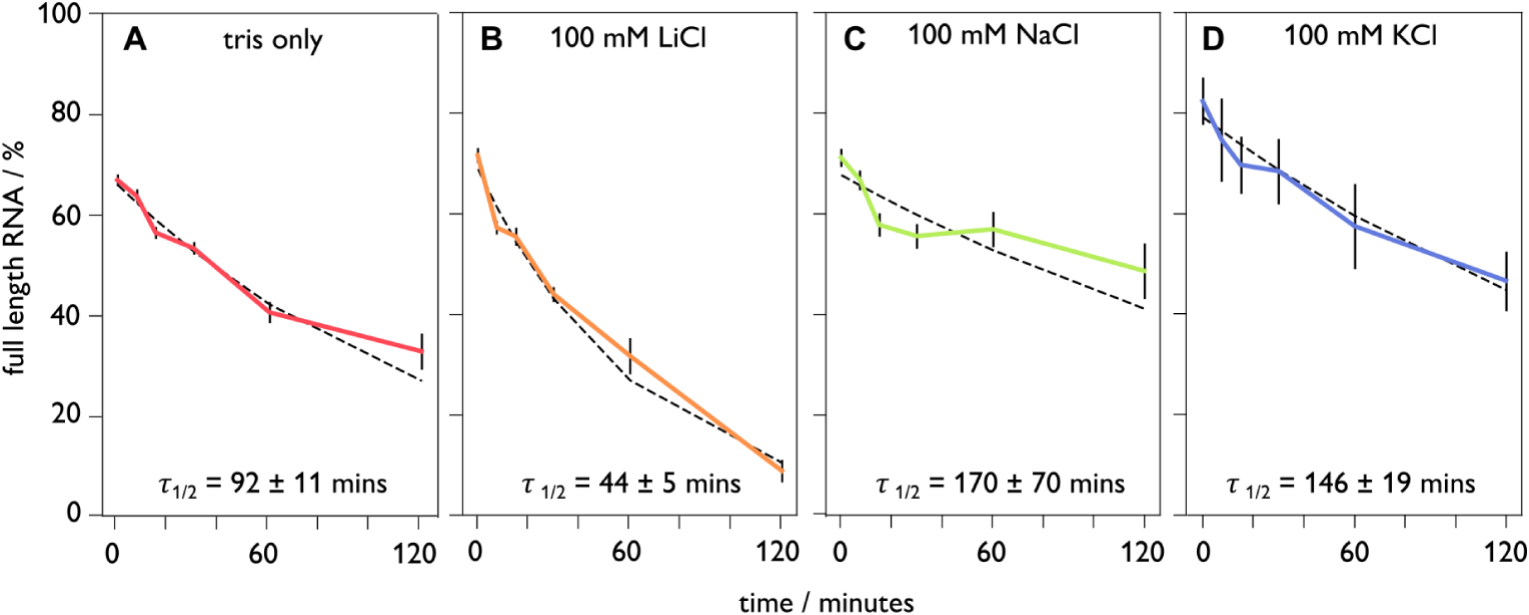
Nanopore-derived degradation profiles of MS2 RNA incubated at 70 °C for varying amounts of time. (A) Tris only, (B) 100 mM LiCl, (C) 100 mM NaCl, (D) KCl. All solutions were buffered at pH 8.0 using 10 mM tris.

While using sodium or potassium results in a similar degradation rate (Figure 3C,D), the use of lithium in the annealing protocol triples the speed with which RNA is cleaved. This finding is in agreement with a previous report,^39^ which showed the cleavage rate for the hepatitis delta virus ribozymes was higher in the presence of lithium than any other monovalent salt tested. It is interesting to note here that another study describing the acceleration of ribozyme activity in lithium relative to sodium suggested that ionic radius was an important factor,^40^ commensurate with our finding that NaCl and KCl exhibit the slowest degradation rates of our tested salt conditions (Figure 3D). To prevent RNA degradation for future analyses, the use of larger monovalent cations such as Na^+^ or K^+^ in annealing protocols may be advisable, rather than Li^+^. It is also interesting to note that even tris buffer alone yields more degradation than Na^+^ or K^+^.

These results for degradation at 70 °C in 10 mM Tris with 100 mM LiCl, NaCl, and KCl were validated by quantitative electrophoresis with an Agilent TapeStation system, for which electropherograms are shown in Figure S7. The integrated fluorescence fraction of the full-length MS2 was calculated after 30 and 120 min in each condition and the half-life calculated (Figure S8). Again, degradation occurred most rapidly in a buffer containing LiCl while NaCl and KCl were approximately equivalent. This confirms the validity of using our nanopore-based method for assessment of RNA integrity.

The half-lives determined by electrophoresis agree with those calculated from the nanopore data and parameter estimation in the case of NaCl and KCl, while the half-life estimated by the nanopore method is significantly shorter in the case of LiCl (44 versus 81 min). This is likely owing to the difference in what is measured in each method; fragments in electrophoresis produce a smaller fluorescence signal, as shorter RNA fragments sequester fewer dye molecules, while the nanopore method counts and sizes fragment molecules directly. As self-cleavage continues and fragments continue to break down into more fragments, this rapidly decreases the proportion of molecules that are close to full length, giving a greater effective decay rate as degradation continues. We expect this effect to be most pronounced for more degraded samples, hence there is a large discrepancy in the measured half-lives for LiCl (the condition with the most degradation) relative to NaCl and KCl.

### RNA Degradation at 94 °C with Monovalent Salts

The procedures described here may also be used to probe the typical high temperature denaturation step in PCR primer annealing and to examine its role in RNA degradation. To this end, we incubated the RNA in 100 mM LiCl, NaCl, or KCl at pH 8.0 in 10 mM tris as previously described, then heated the mixture to 94 °C. The resulting histograms are shown in Figure S9. Nanopore measurements at different time points revealed, for example, a half-life of approximately 7 min for KCl (Figure S10), demonstrating how RNA self-cleavage accelerates at elevated temperatures. A comparison of the 94 and 70 °C data for the differing salts is shown in Figure S11. Our conclusion is that high temperature denaturation steps should be kept as short as possible during primer/probe annealing.

### RNA Degradation with MgCl_2_

While monovalent ions form an ionic atmosphere around nucleic acids to mitigate electrostatic repulsion between phosphate groups, divalent cations are known to stabilize secondary and tertiary structures by binding to specific grooves.^41^ This difference in cation binding leads to the stabilization of alternative RNA conformations and typically leads to more self-cleavage activity in the presence of divalent cations. As expected based on previous reports,^39^ incubation with divalent cations (magnesium, in this case) resulted in significant degradation at all tested time points and temperatures, with the cleavage rate in magnesium being ∼100-fold higher than in lithium. After only 1 min at 70 °C incubation of RNA with MgCl_2_ (100 mM), nearly all RNA in the sample is cleaved, as evidenced by the low event count and the short, shallow events observed, indicating nearly total degradation of the sample (Figure S12). This experimental finding highlights why divalent cations should be avoided during RNA annealing procedures, not only to avoid enzymatic activity, but also to mitigate self-cleavage. Our results underline how nanopore sensing may be used to study RNA degradation under various conditions with minimal sample required.

## Conclusions

In summary, we have presented a single-molecule nanopore assay that enables the direct quantification of degradation for large, structured RNA molecules. The methods described here are suitable for high molecular weight RNAs, which are difficult to study using gel electrophoresis. Unlike electrophoresis, which requires RNA samples to be “hydrodynamically equivalent” for comparison,^17^ the technique presented herein is suited even for highly structured molecules and those that may not easily be denatured with chemical methods. Moreover, solid-state nanopore sensing requires only picogram-scale quantities of analyte, permitting the analysis of RNA cleavage at low concentrations. As such, we foresee that this method may be used to assess the degradation profiles of mRNA vaccines, for example, to compare the effects of nucleoside modifications on in-solution stability.

## Supplementary Material



## Notes

Additional experimental details, materials and methods, data analysis details and theory, distinction of ssRNA species by nanopore, controls, sample matrix measurement, assessment of alternative counting methods, agarose gel comparison, drift of peak current distribution due to end degradation, 94 °C degradation data, degradation in MgCl_2_, event dwell time histogram (PDF)

## &

School of Chemistry, Trinity College Dublin, Dublin 2, Ireland
