# Supplementary material for: Quantifying RNA Degradation with Single-Molecule Nanopore Sensing
Source: Anal Chem. 2025 Oct 23;97(43):23841–7. doi: 10.1021/acs.analchem.5c03019 (PMC12590466; doi:10.1021/acs.analchem.5c03019)
Supplement: Supplementary file 1 [file ac5c03019_si_001.pdf]

Supporting Information

Quantifying RNA Degradation with Single-Molecule Nanopore Sensing

Max K. Earle[#], Mohammed F. Alawami[#], Raluca Elena-Alexii, Simon Brauburger,

Ulrich F. Keyser*, Casey M. Platnich*

[#] These authors contributed equally.

Correspondence to: ufk20@cam.ac.uk, cp769@cam.ac.uk

Cavendish Laboratory, University of Cambridge, CB3 0HE Cambridge, United Kingdom

Contents:

1. Materials
2. RNA heating protocols
3. Native agarose gel electrophoresis
4. Nanopore fabrication
5. Nanopore measurements
6. Nanopore data analysis and model fitting
7. Supplementary figures
8. References

1. Materials.

Glass capillaries (inner diameter 0.2 mm, outer diameter 0.5 mm) were purchased from Sutter Instruments (California, USA). MS2 RNA (3569 nt in length) was purchased from Roche and used without further purification. PDMS used in the fabrication of nanopore chips is Sylgard 184 silicone elastomer kit (The Dow Chemical Company, MI, USA). All water is DEPC-treated nuclease-free water (Merck). Stock 10x tris-HCl buffer (pH 8.0, nuclease-free) was purchased from Fisher Scientific. All buffers were filtered with 0.22 µm Millipore syringe filter units (Merck). RNA sample concentrations were measured using a Thermo Scientific Nanodrop™ 2000 Spectrophotometer.

2. RNA heating protocols.

MS2 RNA was diluted to a final concentration of 0.1 µg/µL in 10 mM tris HCl (pH 8.0) and 100 mM salt (LiCl, NaCl, KCl, $MgCl_2$, or no salt) in nuclease-free water. The sample was then mixed by flicking, microcentrifuged, and placed in a thermocycler (Applied Biosystems™ ProFlex™ PCR System, 3 x 32-well) for incubation.

a) 94 °C protocol: The PCR thermocycler was pre-heated to 94 °C, then each sample was incubated for the given time interval (1-5 min), and then immediately put on ice. A control was kept on ice until ready to proceed with the measurement.
b) 70 °C protocol: The PCR thermocycler was pre-heated to 70 °C, then each sample was incubated at 70 °C for the given time interval (7.5, 15, 30, 60, or 120 minutes). Following incubation, RNA samples were immediately put on ice, with a control was kept on ice until ready to proceed with the measurement.

3. Native agarose gel electrophoresis.

Agarose gel electrophoresis (0.8% w/v agarose) was conducted using a BioRad Sub-Cell GT electrophoresis cell with 1 x TBE (with 0.05% sodium hypochlorite solution) as the running buffer. Gels were run for 2.5 hours at 70 V on ice. Samples are prepared to 150 ng RNA in 10 uL for loading and consist of the sample, 1 x TBE buffer and 1 x purple loading dye (no SDS). The ladder used is the 1 kB ladder from New England Biolabs. After running, gels were stained using GelRed (Biotium) and imaged using the GelDoc-It™ (UVP). Gel images were processed using Fiji (ImageJ) by inverting the grayscale and subtracting the homogenous background with 100-150 pixels rolling ball.

4. Nanopore fabrication.

Quartz glass capillaries were pulled to the desired diameter (~10-15 nm) using a laser-heated pipette puller (P-2000, Sutter Instrument, California, USA). The parameters used were: HEAT=475, FIL=0, VEL=25, DEL=170, PUL=225. Please refer to the P-2000 manual for the explanation of the parameters. After pulling, nanopore were cut to length and positioned within a custom polydimethylsiloxane (PDMS) chip with 8 pores per chip, which was then plasma bonded (Femto, Diener Electronic, Germany) to a glass slide. PDMS was used to seal the pores within the

chip and baked at 120 °C for 2 hours for curing. After baking, chips are placed in the plasma cleaner for 5 minutes to ensure a hydrophilic surface layer. 1 x TE buffer with 4 M LiCl with a pH of 9.4 (adjusted with LiOH) was then added to the central reservoir as well as the outer chambers.

Samples for nanopore measurements were diluted to ~ 300 pM in 1 x TE, 4 M Li (pH 9.4) and injected into the central reservoir.

5. Nanopore measurements.

An Axopatch 200B (Molecular Devices, CA, USA) was used to perform nanopore measurements. The signal was filtered with an external Bessel filter (Frequency Devices) at 50 kHz and digitized at a 1 MHz sampling rate with a data card (PCI-6251, National Instruments). Two Ag/AgCl electrodes were prepared by curing 1-mm Ag wires in a 10% solution of NaClO. These were then inserted into the central reservoir (cis) and the outer chamber (trans) to create an electrical circuit across the nanopore. Current-voltage curves were measured from -600 mV to 600 mV to estimate nanopore size prior to measurements. Approximate diameters were calculated from their conductance as previously described. Pores with a maximum current of ~ 10 nA and a root-mean-square (RMS) noise below 7.5 pA were selected for measurements. Data was collected using an in-house LabVIEW program.

6. Nanopore data analysis and model fitting.

Individual events were extracted from the raw current trace using custom Python software (https://github.com/maxearle/nas2) with a minimum current blockade threshold of 50 pA below the baseline. Extracted events were inspected and erroneous events resulting from measurement artefacts were removed. Duration, mean current blockade, peak current blockade and event charge deficit were calculated for each event.

Due to the finite maximum frequency in the nanopore signal (50 kHz, caused by the external Bessel filter), the nanopore measurement system has a finite response time and events with a dwell time shorter than this will be "chopped". Our digitisation setup has a sampling rate of 1 MHz and therefore a Nyquist frequency of 500 kHz. Current data is thus over-sampled by a factor of 10 and an event lasting 10 μs or longer will not be significantly chopped. As shown in Figure S11, 94% of measured events are longer than this and therefore chopped events do not significantly affect model-fitting.

Peak current values were used to fit the probability distribution:

$$P(x|\{A, \mu, \sigma, k\}) = \frac{A}{\sqrt{2\pi\sigma^2}} e^{-\frac{(x-\mu)^2}{2\sigma^2}} + (1-A)ke^{kx}$$

Where x is the peak current and A, μ, σ and k are parameters to be fit. A corresponds to the total probability contributed by the Gaussian component of the model and is used as an estimate of intactness of the measured sample. μ is the location of the centre of the Gaussian contribution, σ is the standard deviation of the Gaussian contribution and k is the decay constant of the exponential contribution. The function is normalised so that $\int_{-\infty}^{0} P(x)\, dx = 1$, and the

exponential component is scaled by the prefactor $(1 - A)k$ to ensures that the total probability contributed by this component is $(1 - A)$.

Fitting is done by the method of Maximum Likelihood Estimation (MLE). The procedure consists of defining a likelihood function built using the peak current observations that takes the parameters as arguments. The likelihood function gives a value proportional to the probability of a set of parameters, given a set of observations. This is given by:

$$L(\{\theta_i\}|\{x_j\}) = \prod_j P(x_j|\{\theta_i\})$$

Where $\{\theta_i\}$ is the set of parameters of P and $\{x_j\}$ is the set of observations. However, it is often more convenient to use the natural logarithm of this function, the log-likelihood:

$$l(\{\theta_i\}|\{x_j\}) = \sum_j \ln(P(x_j|\{\theta_i\}))$$

The aim of MLE is to find the set of parameters that maximises this function. This was done using the statsmodels package for python (https://www.statsmodels.org), because although a custom implementation of the maximisation procedure is straightforward, the implementation of MLE in statsmodels also calculates the Hessian at the maximum from which can be conveniently calculated standard errors in each of the fitted parameters. By the information equality property of the maximum likelihood estimator, the negative inverse Hessian matrix of the log-likelihood function at its maximum is equal to the Fisher information or the asymptotic covariance matrix. Furthermore, under some reasonable assumptions the maximum likelihood estimator is asymptotically normal. Therefore, the calculated standard errors are symmetric about the mean.[1]

7. Supplementary figures.

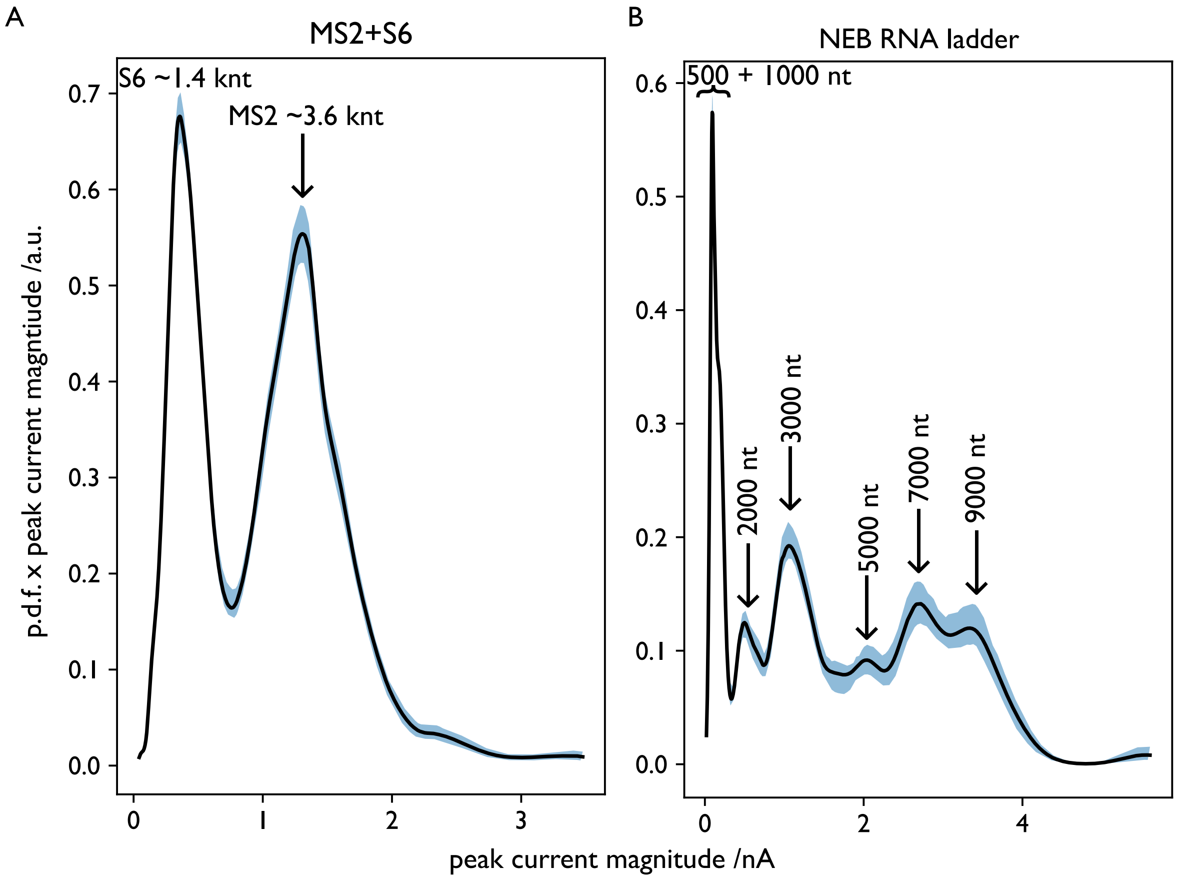

Figure S1. Plots of the weighted estimated probability density function[2] for peak currents versus peak current magnitude in two mixed samples. Different species in the mixed samples can be identified as peaks. Since dyes used in gel electrophoresis label larger molecules more effectively, the p.d.f. is weighted by peak current to increase peak visibility for larger molecules in a similar way to a gel. A) A mixed sample containing fresh MS2 and S6 (another viral RNA) directly from the freezer. B) A ssRNA ladder (New England Biolabs).

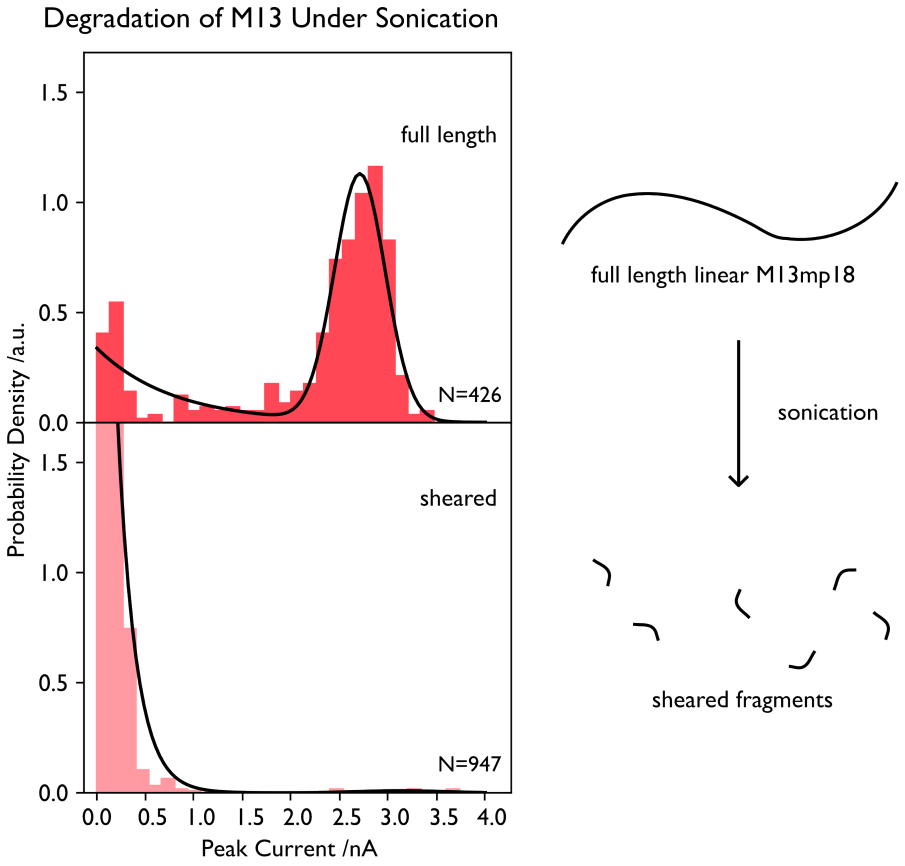

Figure S2. Peak current blockade histograms for a sample of linear M13mp18 before and after shearing via sonication. The full-length sample shows the same dominant Gaussian profile as the untreated MS2 sample used in this work. The sonicated sample shows no trace of Gaussian character but a relative enrichment of very shallow events as in degraded MS2 samples.

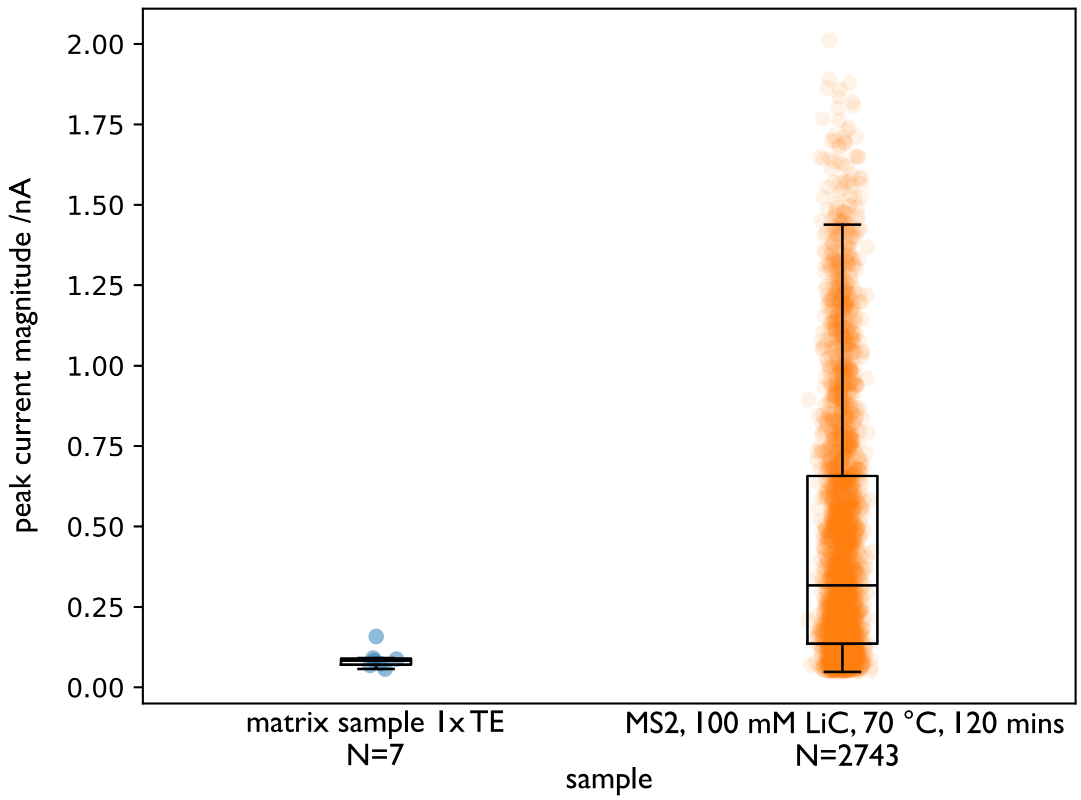

Figure S3. Box and whisker plots for peak current magnitudes in a "matrix sample" consisting of only the 1x TE buffer in which our MS2 is supplied as well as a sample incubated at 70 °C in 100 mM LiCl and 10 mM Tris for 120 minutes. It can be seen that the 1x TE buffer contributes very little background, with only a handful of transient events detected in the absence of an RNA sample.

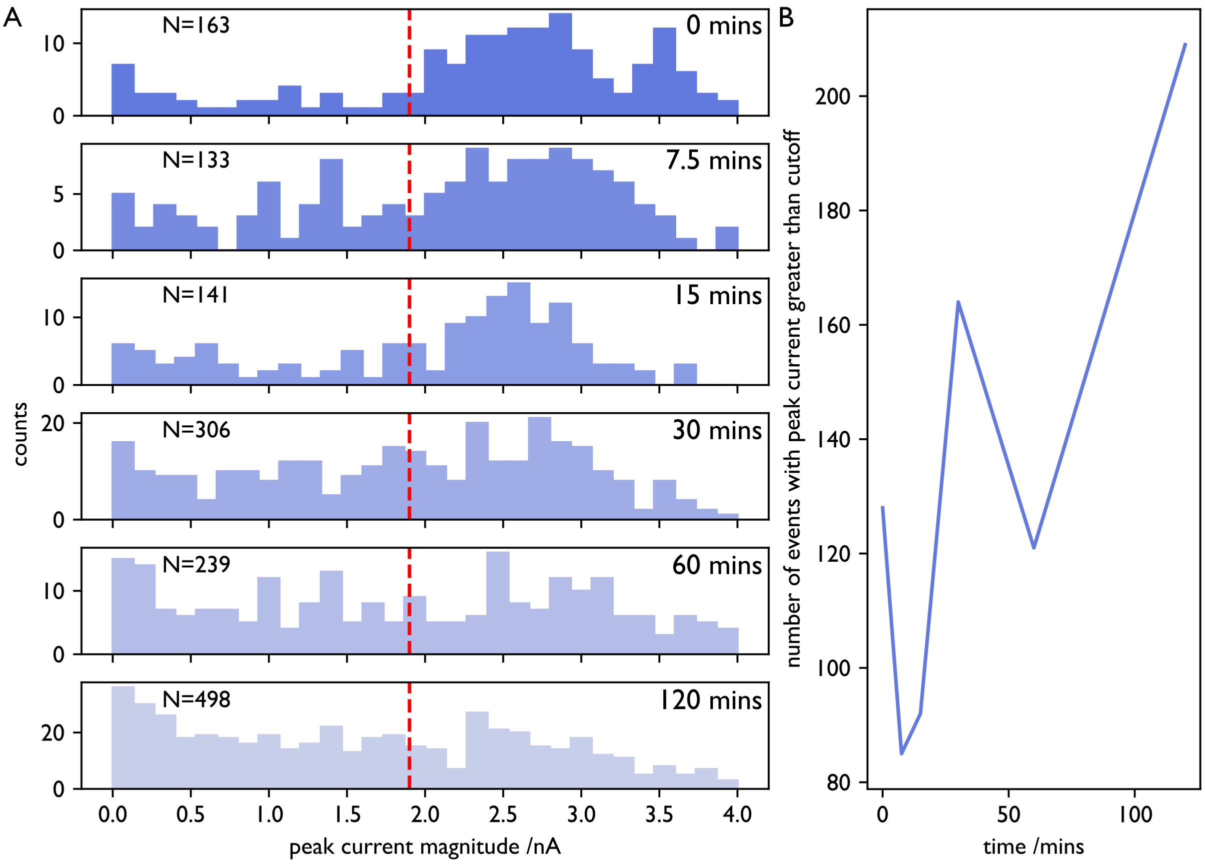

Figure S4. Analysis of degradation of MS2 RNA in 10 mM Tris, 100 mM KCl at 70 °C by counting events above a peak current magnitude threshold (annotated on the histograms in red). A) Peak current magnitude histograms for samples heated to 70 °C between 0 and 120 mins. A peak current threshold is annotated as a red dashed line, above which the intact translocation events lie. B) The number of events above the peak current threshold against time. It can be seen that the number of events above the peak current cutoff does not reflect degradation that is obvious from the peak current magnitude distribution,

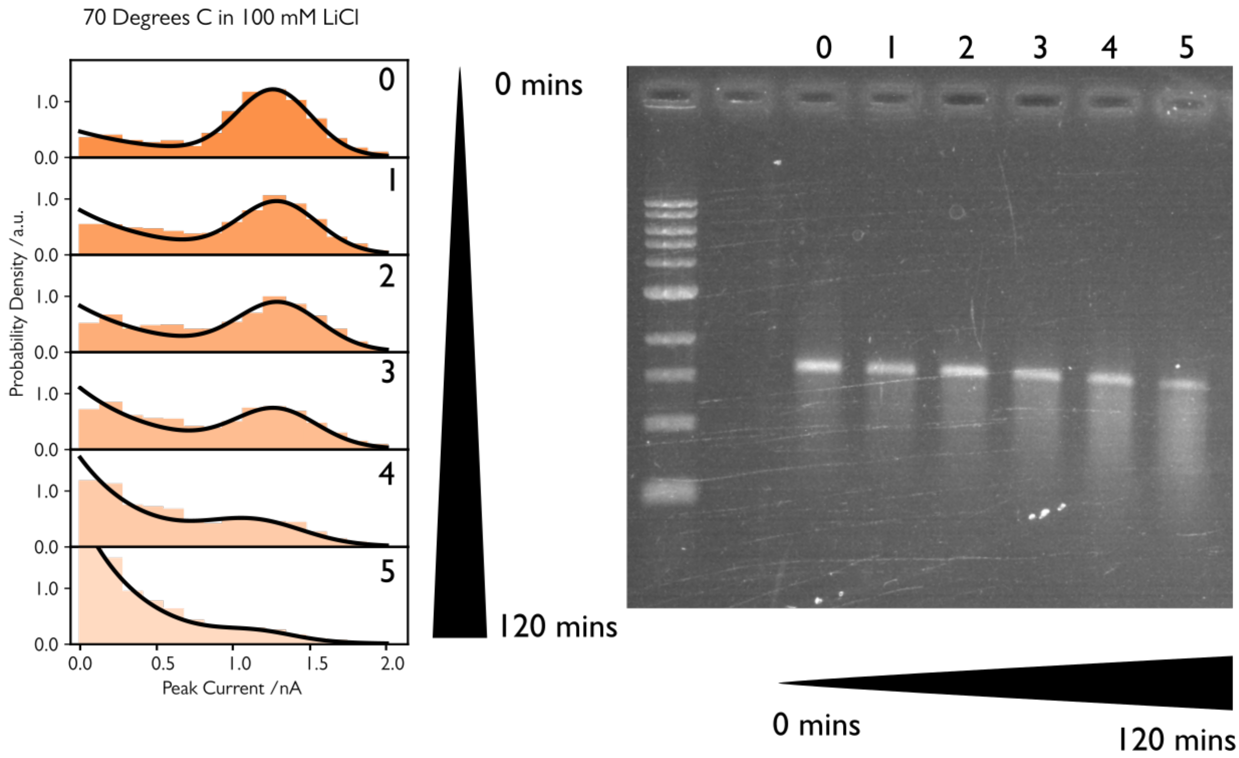

Figure S5. A comparison of MS2 degradation over time as revealed by nanopore analysis versus gel electrophoresis. The two methods both show a reduction in the proportion of intact MS2. The length of the highest molecular weight MS2 population also appears to decrease over time. This is seen in the diminishing of the Gaussian peak and the drift of its centre to smaller values in the nanopore measurement, and in the reduction of main band intensity and increase in band mobility in the gel experiment.

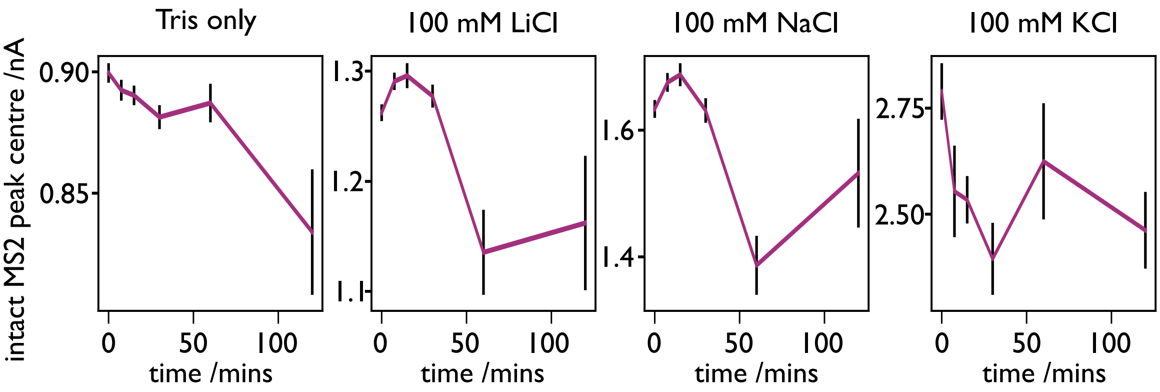

Figure S6. MS2 peak location drift over time at 70 °C, extracted from model fitting in buffer containing 10 mM Tris in addition to: no other salt, 100 mM LiCl, 100 mM NaCl and 100 mM KCl. Peak positions have a tendency to shift to lower peak current values over time, possibly due to degradation from the ends of intact MS2.

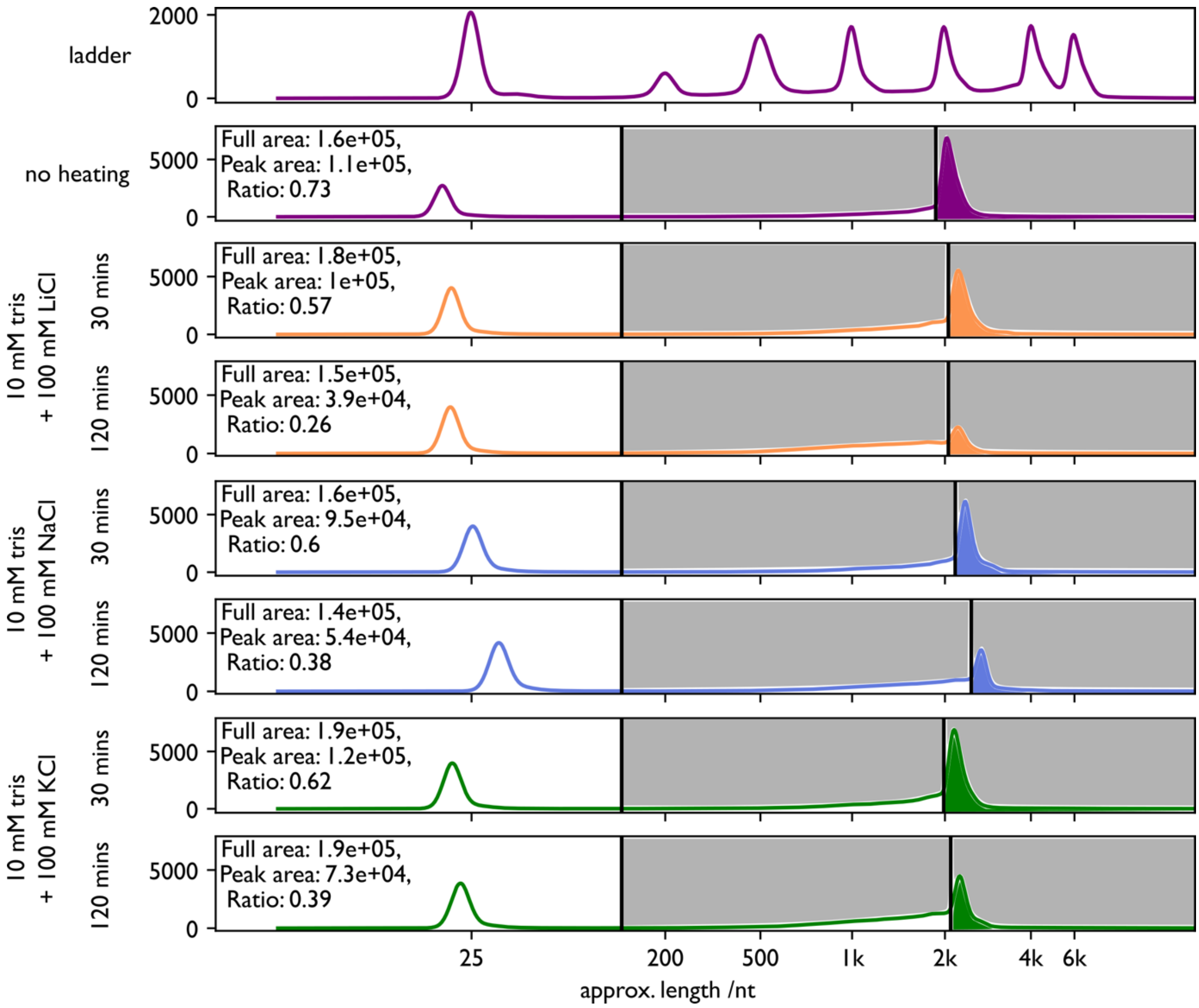

Figure S7. Electropherograms from measurement of degraded MS2 RNA using an Agilent TapeStation system. A ssRNA ladder was measured as well as MS2 RNA directly from a -80 °C freezer and MS2 held at a temperature of 70 °C for 30 and 120 mins in 10 mM Tris with: 100 mM LiCl, 100 mM NaCl and 100 mM KCl. The peak at ~25 nt is produced by an internal standard. For each electropherogram, an integrated fluorescence ratio was calculated by dividing the integrated intensity of the peak corresponding to full-length MS2 (shaded area under the curve) by the integrated intensity of the whole region including the intact peak and the smear of degradation products (region highlighted in grey). 150 ng of MS2 RNA was required for each TapeStation sample, compared to nanopore sensing which can be carried out with as little as 1 picogram of sample.

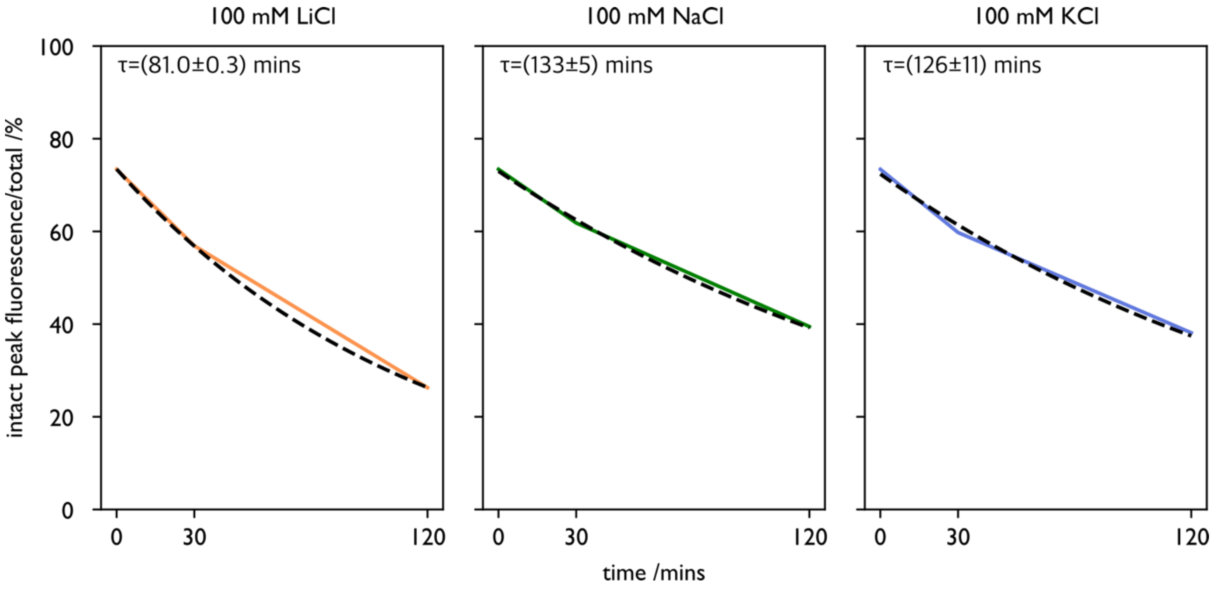

Figure S8. Integrated fluorescence intensity ratios of the peak corresponding to the full-length MS2 from the electropherograms in Figure S7 against time. MS2 was incubated at 70 °C for 30 and 120 minutes in 10 mM Tris with 100 mM LiCl, 100 mM NaCl and 100 mM KCl. The calculated half-lives in each buffer are also shown with the estimated error in the fit. Degradation occurs most rapidly in buffer cont°aining LiCl, with the half-lives in NaCl and KCl being approximately equivalent as in the corresponding nanopore experiments.

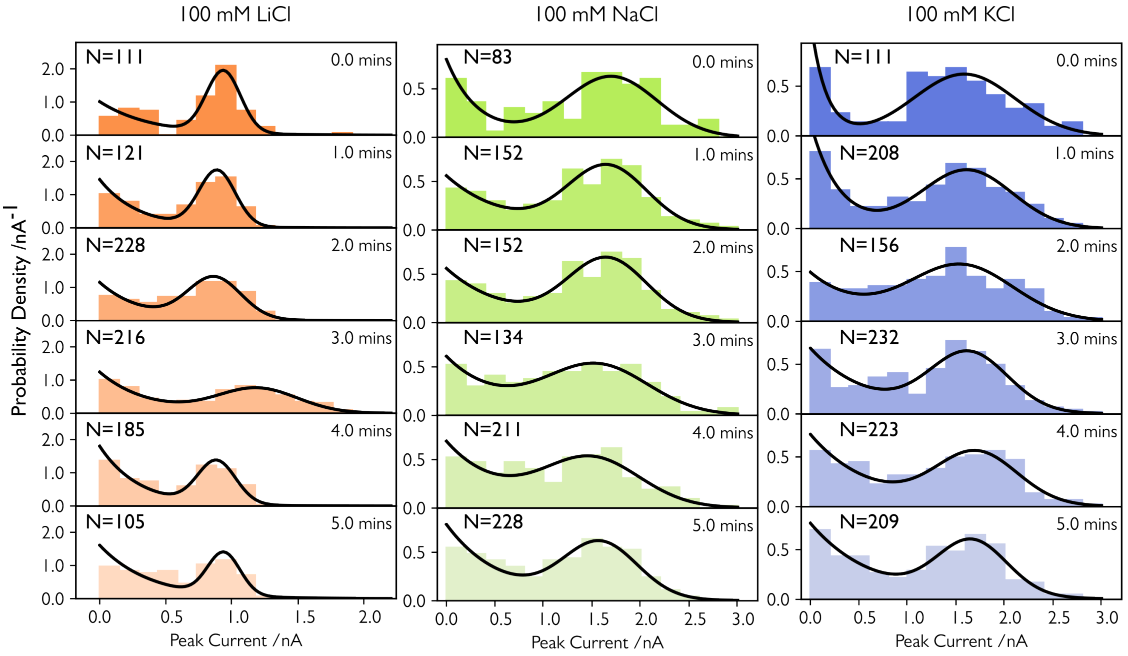

Figure S9. Histograms of single-molecule peak currents from nanopore experiments for MS2 RNA degradation over time. Samples were heated to 94 °C in nuclease-free water with 10 mM tris buffer (pH 8.0) and 100 mM monovalent salt (LiCl, NaCl, or KCl, left to right). With increased heating time, the populations of peak currents shift from the dominant Gaussian (corresponding to the full-length construct) to an exponential tail, indicating degradation.

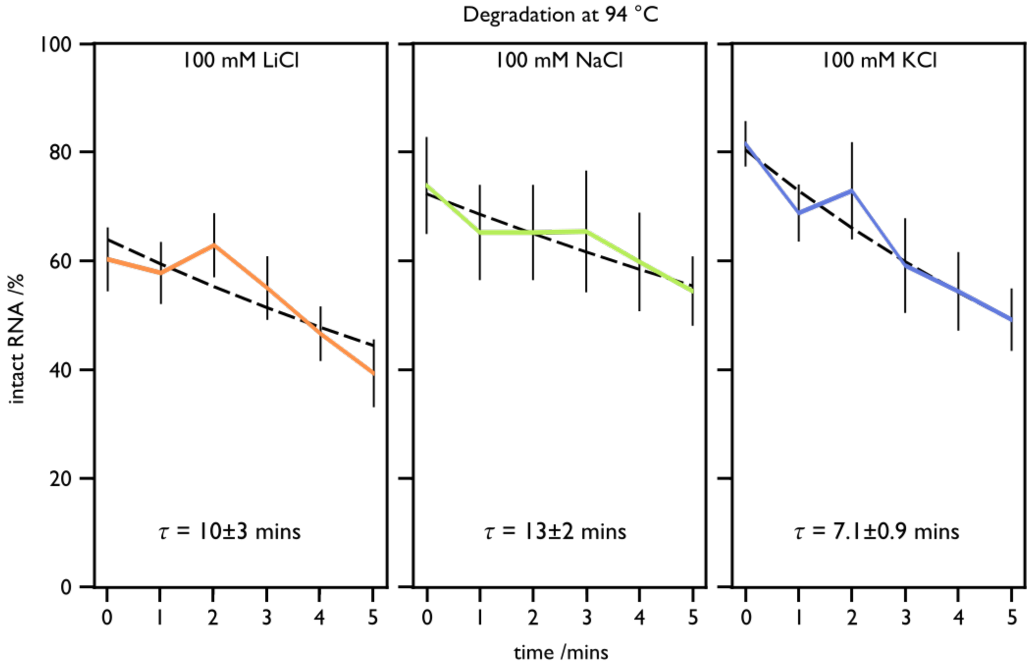

Figure S10. Nanopore-derived degradation profiles of MS2 RNA incubated at 94 °C for varying amounts of time. The half-life τ is also given for each condition. (A) 100 mM LiCl (B) 100 mM NaCl, (C) 100 mM KCl. All solutions were buffered at pH 8.0 using 10 mM tris.

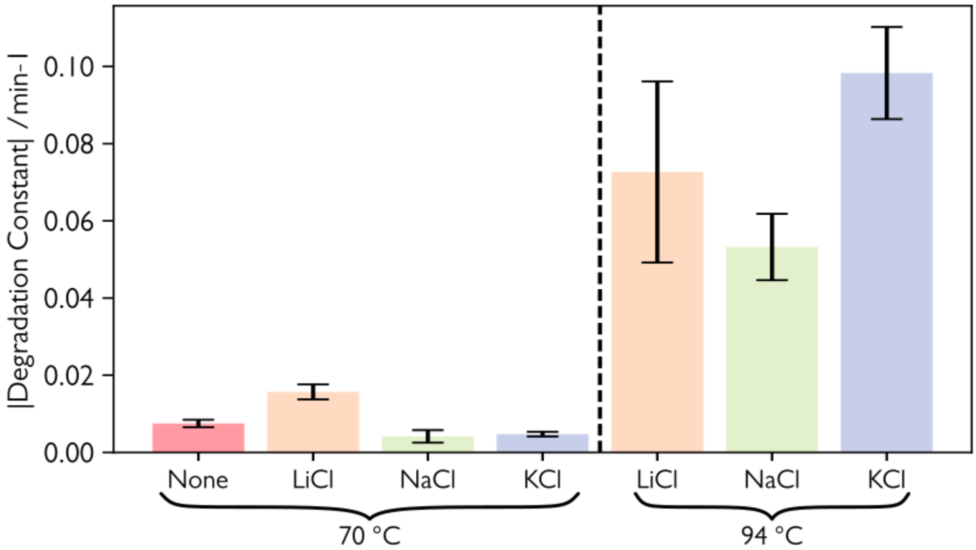

Figure S11. Comparison of degradation rates for MS2 RNA in 10 mM tris buffer (pH 8.0) with either no salt or 100 mM monovalent salt (LiCl, NaCl, KCl) at 70 °C and 94 °C. Degradation rates are up to an order of magnitude greater at 94 °C than at 70 °C, showing the strong effect temperature has on the rate of self-cleavage and the importance of limiting the duration of heating when handling RNA.

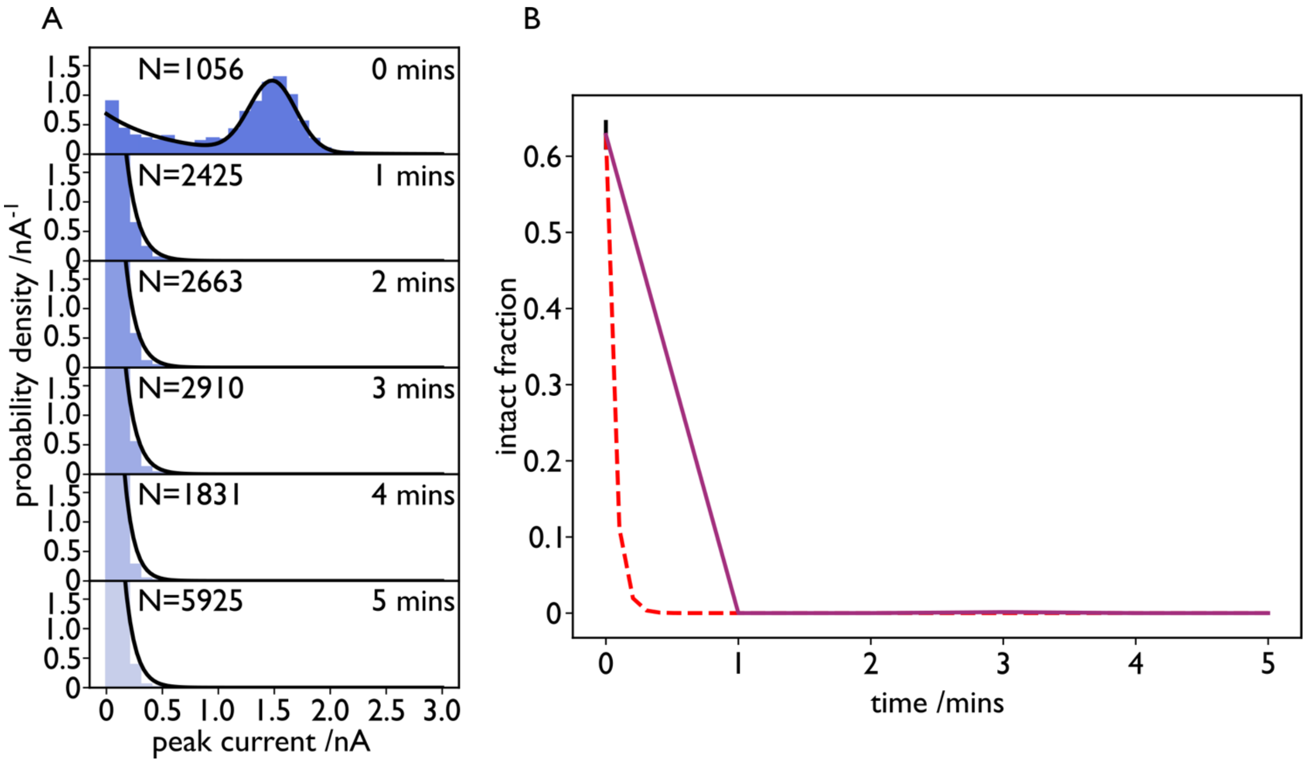

Figure S12. A) Peak current histograms from nanopore measurements of MS2 directly from frozen versus after 1, 2, 3, 4 and 5 minutes of incubation at 70 °C in 100 mM $MgCl_2$ and 10 mM Tris, pH 8.0. The untreated sample shows a dominant population of intact molecules, whilst the treated sample shows no Gaussian peak but instead many shallow events corresponding to degraded molecules. Divalent cations such as Mg^{2+} are known to catalyse self-cleavage of single-stranded RNA. B) Intact MS2 fractions extracted from the model-fitting. Almost all MS2 molecules have substantially degraded in less than 1 minute of heating.

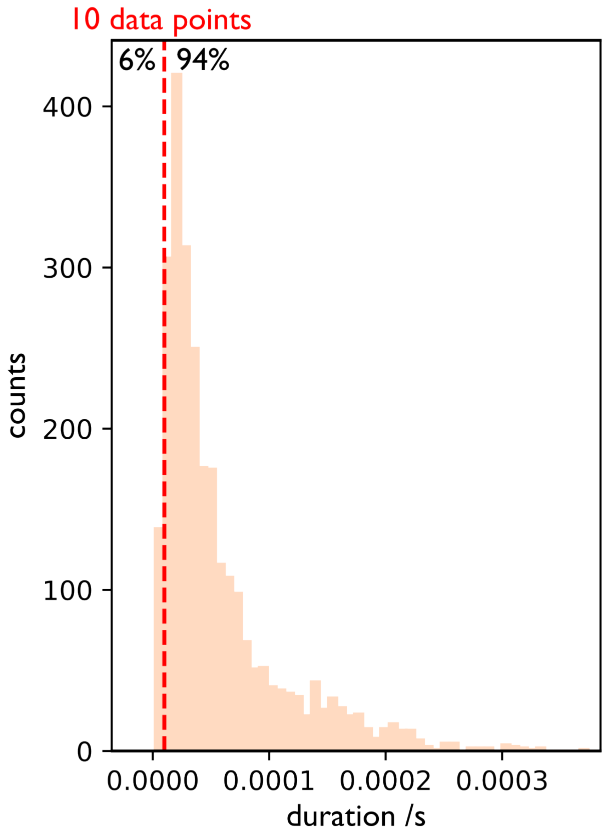

Figure S13. Dwell times for all events in the MS2 sample incubated at 70 °C for 120 minutes in 100 mM LiCl with 10 mM Tris. Although significantly degraded, 94% of events are longer than 10 μs / 10 data points and are therefore not significantly chopped. Thus, chopping does not significantly affect model fitting.

8. References

(1) Newey, W. K.; McFadden, D. Chapter 36 Large sample estimation and hypothesis testing. *Handbook of Econometrics* **1994,** *4*.
(2) Shimazaki, H.; Shinomoto, S.; Shimazaki, H.; Shinomoto, S. Kernel bandwidth optimization in spike rate estimation. *Journal of Computational Neuroscience* **2009** *29*.
